# Active Interventions for the Prevention and Treatment of Pregnancy-Related Lumbopelvic Pain: A Systematic Review and Meta-Analysis

**DOI:** 10.7759/cureus.114589

**Published:** 2026-08-16

**Authors:** Jan Mens, Monique Stikvoort

**Affiliations:** 1 General Practice, Erasmus University Rotterdam, Rotterdam, NLD; 2 Pelvic Girdle Pain, MSK Clinic, Leiden, NLD; 3 Physical Medicine and Rehabilitation, Pelvic Girdle Pain, MSK Clinic, Leiden, NLD

**Keywords:** exercise, low back pain, pelvic girdle pain, pregnancy, systematic review and meta analysis

## Abstract

Lumbopelvic pain (LPP) is common during pregnancy. Reviews of interventions for LPP have generally been inconclusive. We hypothesize that distinguishing between preventive and therapeutic studies, and recognizing that usual care varies between countries, may lead to more robust conclusions. The aim of this study is to determine whether active interventions are effective in preventing or treating LPP during pregnancy, with the ultimate goal of answering the question of which active intervention produces the best outcome. An extensive literature review was conducted to identify studies comparing active interventions with usual care for LPP during pregnancy. Two experienced researchers determined the components of the active intervention: pelvic floor exercises, pelvic stabilizing exercises, or cardiovascular training. Information regarding usual care was gathered from the included studies via a PubMed search, the internet, and through personal contact with experts. The search yielded 33 publications with 38 studies. Pain reduction in all therapeutic non-Scandinavian studies (n = 20) showed statistically significant reductions in pain, with an overall effect size of 2.85 (95% CI 1.36 to 4.34; p < 0.001). In contrast, the effect size of the Scandinavian therapeutic studies (n=2) showed a trend in favor of the control group. For preventive studies, the effect size for pain reduction in the non-Scandinavian studies (n= 6) was 1.10 (95% CI 0.49 to 1.70; p < 0.001), whereas in the Scandinavian studies (n=4) it was 0.06 (95% CI −0.04 to 0.17; p = 0.24). The results regarding disability and prevention of developing sick leave or LPP showed largely the same pattern. No evidence that any specific active intervention was superior to another was found. In summary, studies conducted outside Scandinavia indicate that active interventions are effective for both the treatment and prevention of LPP during pregnancy, whereas studies conducted in Scandinavian countries do not demonstrate such effects. The most plausible explanation for this discrepancy is that active interventions are already incorporated into usual care in Scandinavian countries, resulting in minimal differences between intervention and control groups. From a biomechanical perspective, the successful interventions were highly diverse, suggesting that their effectiveness is unlikely to be explained by biomechanical mechanisms alone. Instead, a common factor across all active interventions appears to be psychosocial in nature. A key element of successful interventions seems to be reassurance, emphasizing that movement-related pain is not harmful to either the mother or the baby. We conclude that active interventions appear to be beneficial for both the treatment and prevention of LPP during pregnancy. The effectiveness of these interventions does not appear to depend on the specific type of active intervention used.

## Introduction and background

Lumbopelvic pain (LPP) is a common condition during pregnancy, with an estimated median prevalence of approximately 45%, although reported rates vary widely [1]. Several hypotheses have been proposed to explain the development and persistence of LPP during pregnancy. The association between increased pelvic joint mobility and LPP has formed the basis of biomechanical theories, which have led to interventions aimed at improving pelvic stability. In contrast, psychosocial models suggest that pain-related anxiety, fear of movement, and reduced confidence in physical activity contribute to pain and disability, supporting interventions that provide information, reassurance, and encouragement to remain active. These different perspectives have resulted in a wide range of interventions, including passive approaches such as pelvic belts and manual therapy, and active interventions ranging from stabilization exercises targeting the deep abdominal and pelvic floor muscles to general exercise programs designed to improve physical fitness. Although many studies report statistically significant effects of active interventions, systematic reviews generally conclude that the evidence is inconsistent [2-5]. Consequently, it remains unclear whether active interventions are truly effective and, if so, which components account for their effectiveness. The contrast between the negative findings of several methodologically high-quality Scandinavian studies and clinical experience, together with our knowledge that usual care in Scandinavian countries generally places greater emphasis on exercise, led us to formulate two hypotheses.

First, we hypothesized that it is important to distinguish between preventive and therapeutic studies. Participants with little or no pain differ substantially in their expectations from those experiencing high pain intensity. Pregnant women with more severe pain, as typically included in therapeutic studies, are primarily interested in strategies to manage and cope with LPP. In contrast, participants with little or no pain at baseline, as commonly included in preventive studies, may become concerned if symptoms emerge or worsen despite the intervention.

Second, we hypothesized that differences in usual care contribute to the inconsistent findings reported in the literature. When comparing active interventions with usual care, it is important to acknowledge that usual care for LPP during pregnancy varies considerably between countries [2].

Taking these two factors into account, we aimed to explore whether the apparent inconsistencies in the literature could be explained, with the ultimate goal of determining whether active interventions are effective in the treatment and/or prevention of LPP during pregnancy.

Although recommendations exist for the classification of pelvic girdle pain (PGP), there is no international consensus on how to distinguish PGP from low back pain (LBP) [6]. Furthermore, RCTs that attempted to distinguish between PGP and LBP using author-defined criteria based on symptoms and physical examination did not demonstrate consistent differences in treatment effects between these conditions. Therefore, no distinction was made in this review between LBP, PGP, or a combination of both. The term LPP was used throughout.

## Review

Methods

Selection of Studies

Relevant studies were searched for in January 2026 in PubMed, CINAHL, Embase, PEDro, and Cochrane, following the guidelines of the PRISMA (Preferred Reporting Items for Systematic Reviews and Meta-Analyses) statement [7]. The search string used in PubMed (Appendix A) was adapted for use in the other databases. In the first round, publications were screened based on their titles and abstracts. In the second round, the full texts were assessed for eligibility. Relevant studies identified from the reference lists of the selected publications were also included. Studies including participants with pain exclusively in the symphyseal region were excluded. Study selection was initially performed by one reviewer. Subsequently, a second reviewer assessed the 38 included studies for eligibility.

Publications in languages other than English were translated using ChatGPT (OpenAI, San Francisco, USA). Translation accuracy was verified by a professional translator for the Iranian publications and by colleagues proficient in Portuguese and Czech for the respective publications. These independent checks did not identify any substantive discrepancies. Studies were included if the effect of an active intervention was compared with usual care. ‘Active intervention’ was defined as supervised exercise, even if the exercises were instructed on only one occasion. Treatment was considered ‘usual care’ if this was explicitly stated in the study or if the authors did not specify the intervention provided to the control group. Providing information on ergonomics and/or activities in addition to usual care was still considered ‘usual care’.

To assess the methodological quality of the studies, we used the Physiotherapy Evidence Database (PEDro) [8].

Mode of Active Interventions

Information was collected on the type of intervention, the frequency of contact with healthcare providers, and whether the exercises were performed individually, in groups, or at home. Exercises were categorized as pelvic floor exercises if the terms “pelvic floor” or “Kegel” were used. Exercises were categorized as pelvic stabilizing exercises if the terms “transverse abdominal” or “deep abdominal” muscles were used. The term “stabilizing exercises” without further specification was not interpreted as pelvic stabilizing exercises. Exercises were classified as cardiovascular fitness if the terms “aerobic”, “cycling”, “running”, “swimming”, “gymnastics”, “Borg rating”, or “heart rate” were used. Two researchers (JM and MS) independently categorized the exercises; disagreements were resolved by a third investigator (PV).

Mode of Usual Care

Information about usual care was gathered through a PubMed search (Appendix B), from the included studies, and through personal communication with experts. The available information consistently indicated that, compared with the other countries included in this review, usual care in the Scandinavian countries (Norway, Sweden, and Denmark) was characterized by more widespread access to physiotherapy and greater emphasis on exercise therapy. Therefore, in the subsequent analyses, the comparison between Scandinavian and non-Scandinavian countries was used as a pragmatic proxy for differences in usual care.

Analysis

If the mean was not reported, the median was used. Standard deviations were estimated by dividing the interquartile range (IQR) by 1.35. When only the range was reported, the SD was estimated as one-quarter of the range. When post-intervention SDs were unavailable but baseline SDs were reported, the pooled baseline SD was used to calculate Cohen’s d, in accordance with the *Cochrane Handbook for Systematic Reviews of Interventions* [9]. Normally distributed continuous variables were summarized as means and standard deviations.

Meta-analyses were presented as forest plots of treatment effects on pain and disability across all studies and, for the preventive studies, of the proportions of participants who developed LPP or took sick leave.

Because pain and disability were assessed using different outcome measures across studies, treatment effects were expressed as standardized mean differences (Cohen’s d), based on the differences between the intervention and control groups at the end of the intervention. This approach allowed results obtained using different measurement scales to be pooled in a single meta-analysis.

Statistical heterogeneity across studies was quantified using the I² statistic. Publication bias was assessed by visual inspection of funnel plots and formally tested using Egger’s regression test.

The association between methodological quality and treatment effect was assessed using a random-effects meta-regression with the PEDro score as a moderator. All analyses were performed using SPSS version 30 (IBM Corp., Armonk, USA). A p-value < 0.05 was considered statistically significant.

Results

**Figure 1:**
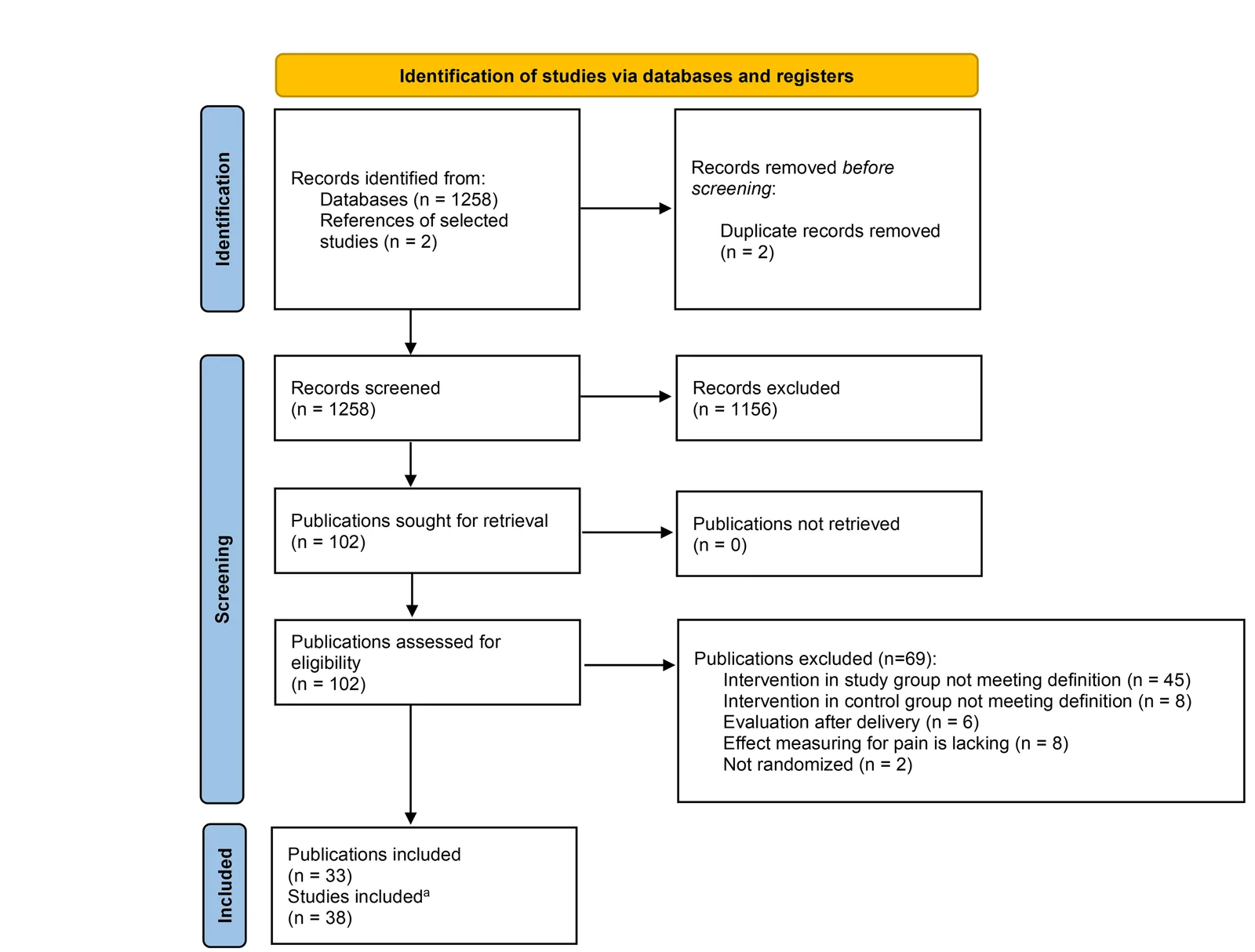
Flowchart according to the PRISMA guidelines. ^a^ Publications with 3 or 4 intervention arms resulted in 2 or 3 studies. PRISMA: Preferred Reporting Items for Systematic Reviews and Meta-Analyses

**Figure 2:**
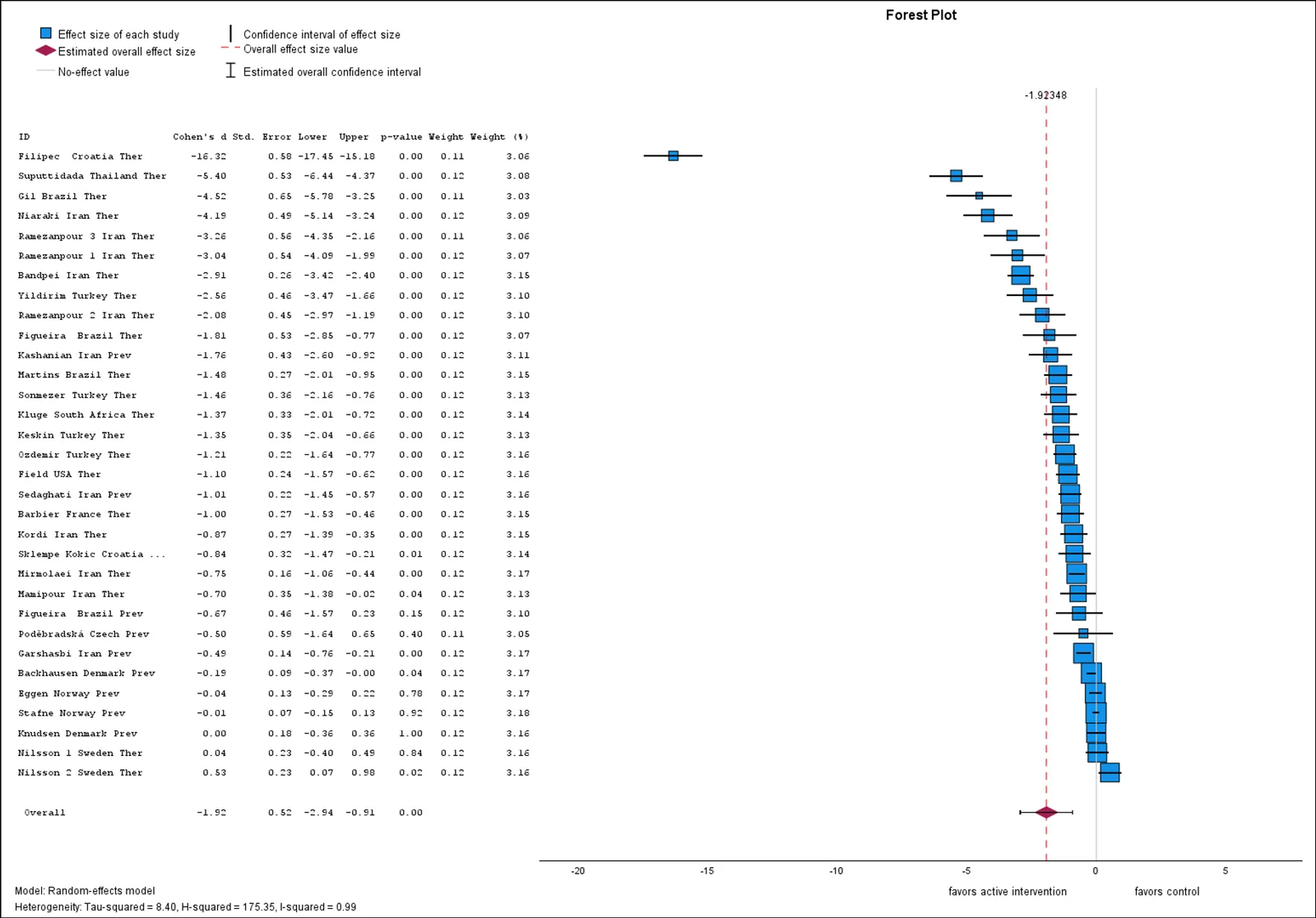
Pain decrease in either therapeutic or preventive studies. References: Table 2, line 4.

The study selection process is presented in Figure 1. Sixteen of the 38 included studies were preventive and 22 were therapeutic (Table 1; Table 2, lines 1-3). A forest plot of pain reduction showed an effect size of 1.92 (p < 0.001) (Figure 2; Table 2, line 4; and Table 3).

**Table 1:** Description of the studies. DRI: Disability Rating Index ; LBP: low back pain; LPP: Lumbopelvic pain; NRS: Numeric Rating Scale; ODI: Oswestry Disability Index; PGP: Pelvic Girdle Pain; PGQ: Pelvic Girdle Questionnaire; QBPDS: Quebec Back Pain Disability scale; RMDQ: Roland Morris Disability Questionnaire; SF: Short Form Health Survey; VAS: Visual Analogue Scale a Not used in the analyses. b The values of patients with LBP and PGP were summed and referred to as LPP in the analyses. c Not used in the analyses because the data were not presented in detail.

First author with year of publication	Country	Preventive (Prev) or therapeutic (Ther)	Most important inclusion criteria	Most important exclusion criteria	Interventions in the experimental group	Intervention in the control group as worded by the authors	Measurements
Backhausen 2017 [10]	Denmark	Prev	age >18 years; 20 weeks pregnant; singleton pregnancy; healthy	BMI>29; severe PGP; abuse problems	exercises in a swimming pool; twice a week for 12 weeks; supervised in the first contact	standard prenatal care	- pain intensity on an NRS - RMDQ - proportion of women with LPP - proportion of women on sick leave
Bandpei 2010 [2]	Iran	Ther	12-22 weeks pregnant; healthy	specific causes of low back pain	exercises to strengthen the abdominal and back muscles; daily for 12 weeks; supervised in the first contact; ergonomic advice	no intervention	- pain intensity on a VAS - ODI
Barbier 2023 [11]	France	Ther	15-32 weeks pregnant; singleton pregnancy; healthy; BMI< 32 kg/m^2^	specific causes of low back pain; high-risk pregnancy	stretching and contraction of paravertebral muscles; 20-30 minutes, 2-3 times a week for two months	advice on ergonomics and activities	- pain intensity on an NRS - physical component SF-12
Eggen 2012 [6]	Norway	Prev	age 18-40 years; 20 weeks pregnant; singleton pregnancy; healthy	high-risk pregnancy	aerobics, activation of stabilizing muscles and pelvic floor; once a week in classes supervised and daily at home 16-20 weeks; ergonomic advice	standard care	- pain intensity morning on an NRS^a^ - pain intensity evening on an NRS - RMDQ - physical component SF-8^a^ - proportion of women with PGP^b^ - proportion of women with LBP^b^
Field 2012 [12]	USA	Ther	age 18-40 years; 18-22 weeks pregnant; singleton pregnancy; depressed; otherwise, healthy	drug abuse; other psychiatric condition than depression	yoga; 20 minutes in classes, once a week, from inclusion to week 32	standard prenatal care	- pain intensity on an NRS
Figueira 2014 [13]	Brazil	Prev	age >18 years; 20-31 weeks pregnant; healthy	high-risk pregnancy	static flexibility exercises 45 minutes; twice a week for 9 weeks	conventional medical treatment with postural guidelines for LBP prevention	- pain intensity on a VAS
Figueira 2014 [13]	Brazil	Ther					
Filipec 2020 [14]	Croatia	Ther	age 25-45 years; 10-34 weeks pregnant	other causes of back pain	pelvic tilt exercises, gluteus contractions and active hip flexion; 20 minutes two times a week	the control group continued to adhere to their regular life habits.	- pain intensity on a VAS - QBPDS
Garshasbi 2005 [15]	Iran	Prev	age 20-28 years; 17-22 weeks pregnant; healthy; nulliparous; housewife; high school graduated	severe back pain before pregnancy; orthopedic diseases;	strengthening of trunk and hip muscles; cardiovascular fitness; three times a week for 12 weeks;	not mentioned	- Pain intensity with a modified QBPDS
Gil 2011 [16]	Brazil	Ther	age 18-40 years; 20-25 weeks pregnant; nulliparous	pathology of the spine; obstetric or gynecologic pathology	stretching by means of Global Postural Re-education; 40 minutes, once a week for 8 weeks	routine prenatal care	- pain intensity with a VAS - RMDQ 24
Haakstad 2015 [3]	Norway	Prev	<24 weeks pregnant; singleton pregnancy; nulliparous; healthy; sedentary	inability to attend weekly exercise classes; insufficient knowledge of the Norwegian language	as control group and aerobic dance with focus on endurance, strength of stabilizing muscles and pelvic floor; 2-3 times a week for 12 weeks; supervised; ergonomic advice	usual prenatal care	- proportion of women with PGP - proportion of women with LBP^b^ - questions about influence of LPP on activities of daily life (Y/N) and about influence of LPP on physical activity (Y/N)^a^
Kashanian 2009 [17]	Iran	Prev	age 20-30 years; 16 weeks pregnant; nulliparous; healthy	drug abuse	as control group and relaxation and strengthening of various muscles of trunk, shoulder and hips; three times a week for 8 weeks	routine prenatal care	- pain intensity (method not mentioned)
Keskin 2012 [18]	Turkey	Ther	>32 weeks pregnant	other causes of back or pelvic pain	pelvic tilt exercises, stretching muscles of lower extremity, isometric abdominal contractions and posture exercises; two times a day for 3 weeks	not mentioned	- pain intensity with a VAS - RMDQ 24
Kihlstrand 1999 [19]	Sweden	Prev	<19 weeks pregnant; healthy	epilepsy; insufficient knowledge of the Swedish language	water-based exercises; once a week for 12-17 weeks; supervised	not mentioned	- pain intensity at its worst on a VAS^c^ - pain intensity at its mildest on a VAS^c^ - pain intensity ‘just now’ on a VAS^c^ - proportion of women with LPP - proportion of women on sick leave because of LPP
Kluge 2011 [20]	South Africa	Ther	age 20-40 years; 16-24 weeks pregnant	pathology of the spine; obstetric or gynecologic pathology	exercises to strengthen and activate transverse abdominal muscles, pelvic floor, hip muscles and quadriceps; daily at home and every 2 weeks 30-45 minutes in supervised classes for 10 weeks	basic care and information about ergonomics and correct posture	- pain intensity on NRS 0-60 - Likert-Modified RMDQ 0-144
Knudsen 2024 [21]	Denmark	Prev	<15 weeks pregnant; sedentary; healthy		as control group and exercises in gym or swimming pool; supervised; once a week from inclusion to delivery	standard prenatal care	- pain intensity - physical component SF-36 - proportion of women on sick leave with medical certificate - proportion of women on sickness absence without medical certificate^a^ - proportion of women with LPP
Kordi 2013 [22]	Iran	Ther	age <40 years; 20-32 weeks pregnant; singleton pregnancy; healthy; positive tests for PGP	contraindications to exercise in pregnancy	as control group and hip, trunk, and pelvic floor muscles, two times a day, three times a week for 6 weeks	general information	- pain intensity on a VAS - ODI
Mamipour 2023 [23]	Iran	Ther	age >18 years; 17-18 weeks pregnant; nulliparous; healthy; LPP for more than one week	history of LPP before pregnancy	as control group and activating pelvic floor and deep abdominal muscles; three times a week for 10 weeks; in supervised groups and at home;	standard antenatal care and customary information	- pain intensity on a VAS - ODI
Martins 2005 [24]	Brazil	Ther	>12 weeks pregnancy; singleton pregnancy; living in Paulínia	contraindication for training; already treated with physiotherapy	stretching by means of Global Postural Re-education; one hour, once a week for 8 weeks	treatment according to medical guidelines	- pain intensity on a VAS
Miquelutti 2013 [25]	Brazil	Prev	age 16-40 years; ≥18 weeks pregnant; singleton pregnancy; healthy		stretching of various muscle groups, activating the pelvic floor and transverse abdominal muscles; aerobics; breathing techniques and relaxation exercises in preparation for childbirth; daily at home and supervised every 1-4 weeks	routine of prenatal care	- proportion of women with LPP
Mirmolaei 2018 [4]	Iran	Ther	age 18-35 years; 17-22 weeks pregnant; singleton pregnancy; healthy	contraindications to exercise	as control group and ergonomic advice; stretching and strengthening muscles; 10 times a day for 12 weeks at home	routine prenatal care	- pain intensity on a VAS - ODI
Mørkved 2007 [5]	Norway	Prev	18 weeks pregnant; nulliparous; healthy		as control group and aerobic training, activation of stabilizing muscles and pelvic floor; weekly training at home twice daily for 12 weeks	customary information and providing a pelvic belt	- DRI - proportion of women on sick leave - proportion of women with LPP
Niaraki 2021 [26]	Iran	Ther	age 20-35 years; 20-26 weeks pregnant; singleton pregnancy; RMDQ 24 score ≥12; VAS Pain ≥4	contraindications to exercise; known musculoskeletal, neurological, or psychiatric disorder; use of painkillers or fear of water	aerobics in water and stretching 60 minutes, three times a week for 2 months	standard prenatal care	- pain intensity with a VAS - RMDQ 24
Nilsson 2005 [27]	Sweden	Ther	<35 weeks pregnant; three positive tests for PGP	positive tests for LBP	home exercises of muscle among hips and balance training; two times a week for 16 weeks; supervised in the first contact	customary information and providing a pelvic belt; availability to call a helpdesk.	- pain intensity on a VAS median - DRI
Nilsson 2005 [27]	Sweden	Ther			exercises for muscle strength and cardiovascular fitness two times a week for 16 weeks; all supervised	customary information and providing a pelvic belt; availability to call a helpdesk.	
Ostgaard 1994 [28]	Sweden	Prev	18 weeks pregnant; nulliparous; healthy		Supervised individual sessions with back school education and training: 5 sessions of 30 minutes and training at home three times a week; providing general information and ergonomic advice; half of the women with LPP were given a pelvic belt	according to the usual routines	- pain intensity^c^ - proportion of women on sick leave - proportion of women with LPP^c^
Ostgaard 1994 [28]	Sweden	Prev			supervised classes with back school education and training 2 sessions of 45 minutes; providing general information and ergonomic advice; half of the women with LPP were given a pelvic belt	according to the usual routines	
Ozdemir 2015 [29]	Turkey	Ther	age >18 years; 20-35 weeks pregnant; sedentary lifestyle	severe low back and/or pelvic disease before pregnancy; using other treatments for low back or pelvic pain	stretching, strengthening, and loosening of spine muscles on a mattress or brisk walking; 30 minutes, three times a week for 4 weeks	usual care	- pain intensity with a VAS - ODI
Poděbradská 2019 [30]	Czech	Prev	end of second trimester or beginning of third trimester of pregnancy		exercises to reduce the pronation position of the feet; daily 10-15 minutes in the second trimester of the pregnancy	no specific exercises	- McGill Pain Questionnaire - Pregnancy Mobility Scale
Ramezanpour 2018 [31]	Iran	Ther	age >24 years; 20-35 weeks pregnant; primigravida; singleton pregnancy; healthy	exercises before pregnancy	body balance three times a week for 12 weeks	usual care	- pain intensity on a VAS
Ramezanpour 2018 [31]	Iran	Ther			pelvic floor exercises; two times a day for 12 weeks	usual care	
Ramezanpour 2018 [31]	Iran	Ther			body balance three times a week and pelvic floor exercises; two times a day for 12 weeks	usual care	
Sedaghati 2007 [32]	Iran	Prev	not mentioned	contraindications to aerobics	supervised aerobics 60 min with accent on bicycling, 3 times a week	not mentioned	- pain intensity with a modified QBPDS
SklempeKokic 2017 [33]	Croatia	Prev	age 20-40 years; <30 weeks pregnant	contraindications for exercise, smoking, severe lumbopelvic pain before pregnancy	aerobics, brisk walking; resistance exercises of muscles in the lumbopelvic area, upper and lower limb, back extensors, pelvic floor and deep abdominal muscles; two times a week from inclusion to end of pregnancy	standard antenatal care	- pain intensity with an NRS - RMDQ 24 - PGQ^a^
Sonmezer 2021 [34]	Turkey	Ther	age 20-35 years; 22-24 weeks pregnant; parity ≤3	pre-pregnancy lumbar pain; contraindications for exercises	Pilates exercises 60 minutes; two times a week for 8 weeks	regular prenatal care	- pain intensity on a VAS - ODI
Stafne 2012 [35]	Norway	Prev	18-22 weeks pregnant; singleton pregnancy; healthy	high-risk pregnancies	supervised aerobics; activation of stabilizing muscles and pelvic floor; 60 minutes once a week; providing ergonomic advice	standard antenatal care; providing general information	- pain intensity in the morning^a^ - pain intensity in the evening - DRI - proportion of women on sick leave - proportion of women with LPP
Suputtidada 2002 [36]	Thailand	Ther	not mentioned	not mentioned	sitting pelvic tilt exercises; 4-10 repetitions per session, two times a day, five days per week, for 8 weeks	did not perform any exercise	- pain intensity on a VAS
Yildirim 2023 [37]	Turkey	Ther	age 18-40 years; 14-24 weeks pregnant; singleton pregnancy; healthy; VAS pain ≥2	other cause of back pain; contraindications for exercises	pilates exercises; 60 minutes, two times a week for 12 weeks	usual prenatal care	- pain intensity on a VAS - RMDQ 24

**Table 2:** References of different selections of studies. ^a^ Some publications include two or three separate studies.

Line number	Study selections	References
1	All studies	[2-6, 10-37]^a^
2	All therapeutic studies	[2,4,11-14,16,18,20,22-24,26,27,29,31,34,36,37]^a^
3	All preventive studies	[3,5,6,10,13,15,17,19,21,25,28,30,32,33,35]^a^
4	Therapeutic and preventive studies with numeric data on pain reduction	[2,4,6,10-18,20-24,26,27,29-37]^a^
5	Therapeutic studies with significant pain reduction	[2,4,11-14,16,18,20,22-24,26,29,31,34,36,37]^a^
6	Therapeutic studies without significant pain reduction	[27]^a^
7	Therapeutic studies with numeric data on disability reduction	[2,4,11,14,16,18,20,22, 23,26,27,29,34,37]^a^
8	Preventive studies with numeric data on pain reduction	[6,10,13,15,17,21,30,32,33,35]
9	Preventive studies without numeric data on pain reduction	[3,5,19,25,28]^a^
10	Preventive studies without significant pain reduction	[3,5,6,13,19,21,25,28,30,35]
11	Preventive studies with numeric data on disability reduction	[6,10,21,30,35]
12	Sick leave percentages in preventive studies	[3,5,10,19,21,28,35]^a^
13	Lumbopelvic pain percentages in preventive studies	[3,5,6,10,19,21,25,35]
14	Scandinavian studies without significant pain reduction	[5,6,19,21,27,28,35]^a^
15	Scandinavian study with a significant pain reduction	[10]

Therapeutic Studies

**Figure 3:**
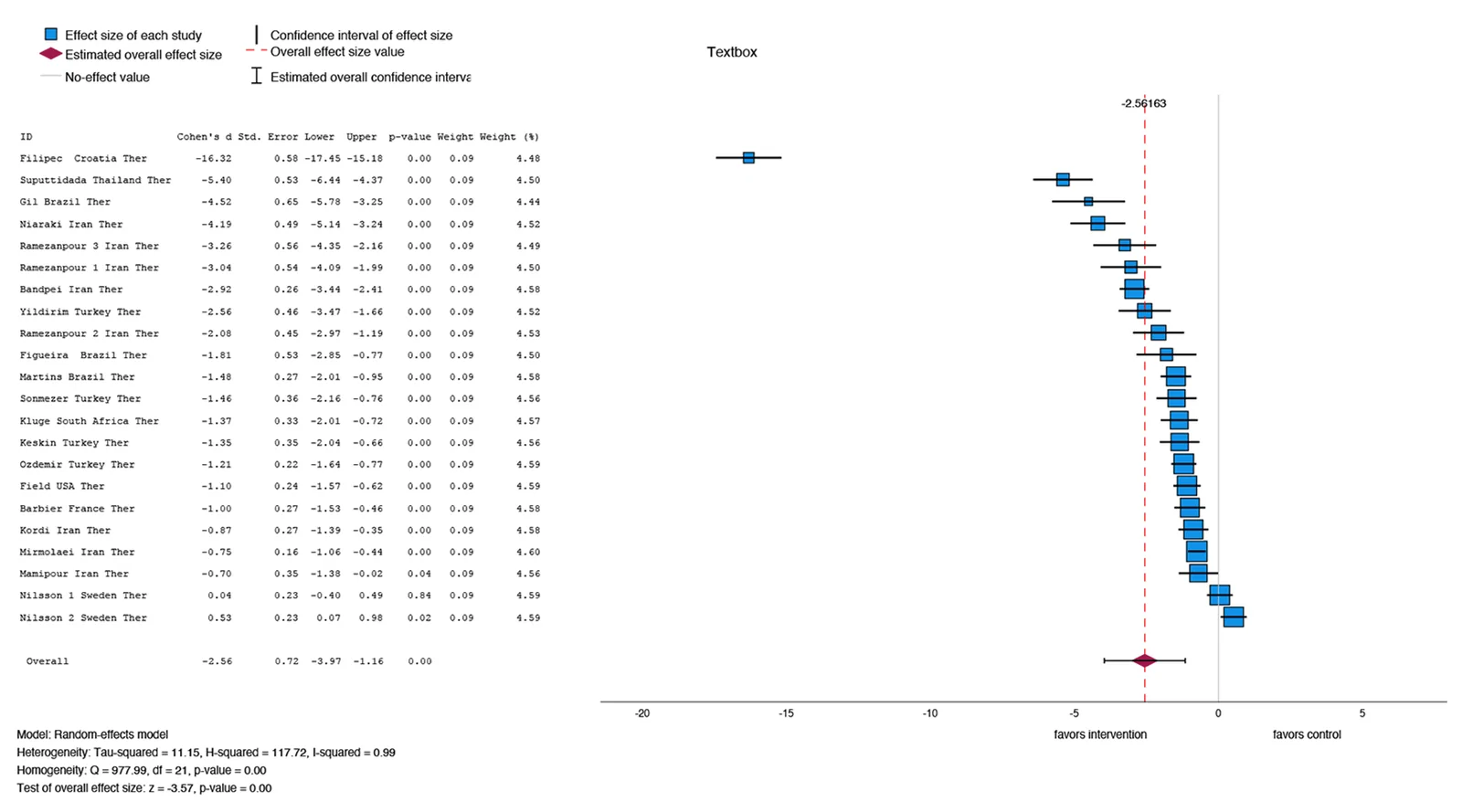
Pain decrease in therapeutic studies. References: Table 2, line 2.

The effect size for pain reduction in the 22 therapeutic studies was 2.56 (p < 0.001) (Table 2, line 2; Table 3; Figure 3). Twenty of these studies showed a statistically significant reduction in pain (Table 2, line 5; Figure 3). The two studies without a statistically significant effect were the two Scandinavian studies (Table 2, line 6). The 20 therapeutic studies conducted outside Scandinavia showed an overall effect size for pain of 2.85 (p < 0.001) (Table 3). In contrast, the effect size of the two Scandinavian therapeutic studies was negative (−0.28), indicating a trend in favor of the control group (Table 3).

**Table 3:** Effect sizes and I-square of different selections of studies.

Figure number in current article	Number of studies	Study category	I^2^	Effect size (95% CI)	p-value
		All studies			
2	32	Pain decrease	0.99	1.92 (0.91 to 2.94)	<0.001
		Therapeutic studies			
3	22	Pain decrease in all countries	0.99	2.56 (1.16 to 3.97)	<0.001
	20	Pain decrease without Scandinavia	0.99	2.85 (1.36 to 4.3)	<0.001
	2	Pain decrease only in Scandinavia	0.55	-0.28 (-0.76 to +0.19)	0.24
4	15	Disability decrease in all countries	0.99	2.30 (0.90 to 3.72)	0.001
	13	Disability decrease without Scandinavia	0.99	2.64 (1.10 to 4.18)	<0.001
	2	Disability decrease only in Scandinavia	0.20	0.13 (-0.23 to +0.48)	0.49
		Preventive studies			
5	10	Pain decrease in all countries	0.89	0.46 (0.14 to 0.77)	0.004
	6	Pain decrease without Scandinavia	0.51	0.84 (0.49 to 1.19)	<0.001
	4	Pain decrease only in Scandinavia	0.11	0.065 (-0.042 to +0.17)	0.23
6	5	Disability decrease in all countries (only one study outside Scandinavia)	0.00	0.069 (-0.029 to +0.16)	0.17
7	8	Sick leave percentage in all countries (only Scandinavian studies)	0.48	0.16 (-0.17 to +0.49)	0.34
8	8	LPP percentage in all countries (only one study outside Scandinavia)	0.23	0.15 (-0.094 to +0.39)	0.23

**Figure 4:**
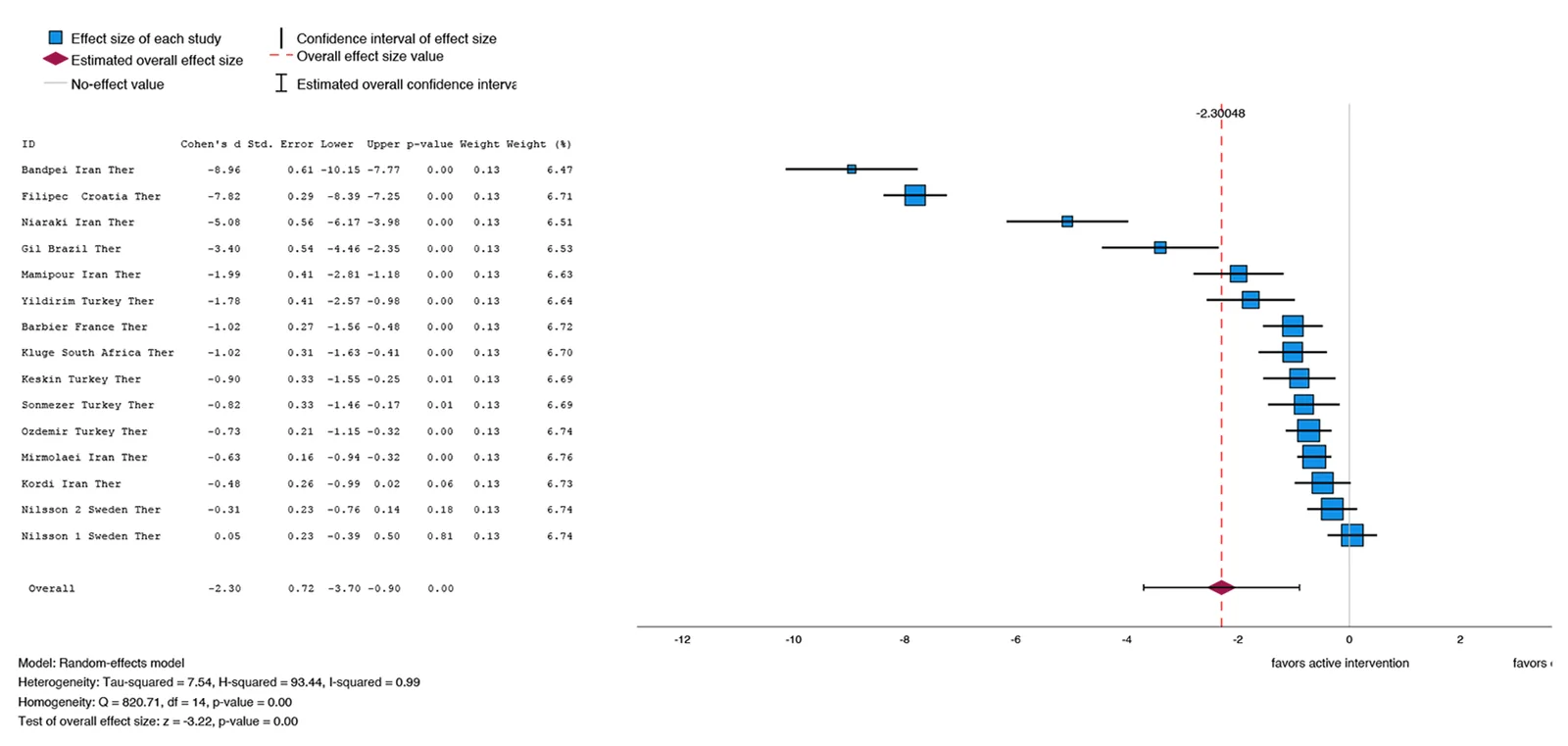
Disability decrease in therapeutic studies. References: Table 2, line 7.

Of the 15 therapeutic studies that reported results for disability, 12 showed a statistically significant effect (Table 2, line 7; Table 3; Figure 4). The two Scandinavian studies were among the three exceptions. The overall effect size for disability across the 15 studies was 2.30 (p = 0.001) (Table 3). The 13 studies conducted outside Scandinavia showed an overall effect size for disability of 2.64 (p < 0.001) (Table 3).

Preventive Studies

**Figure 5:**
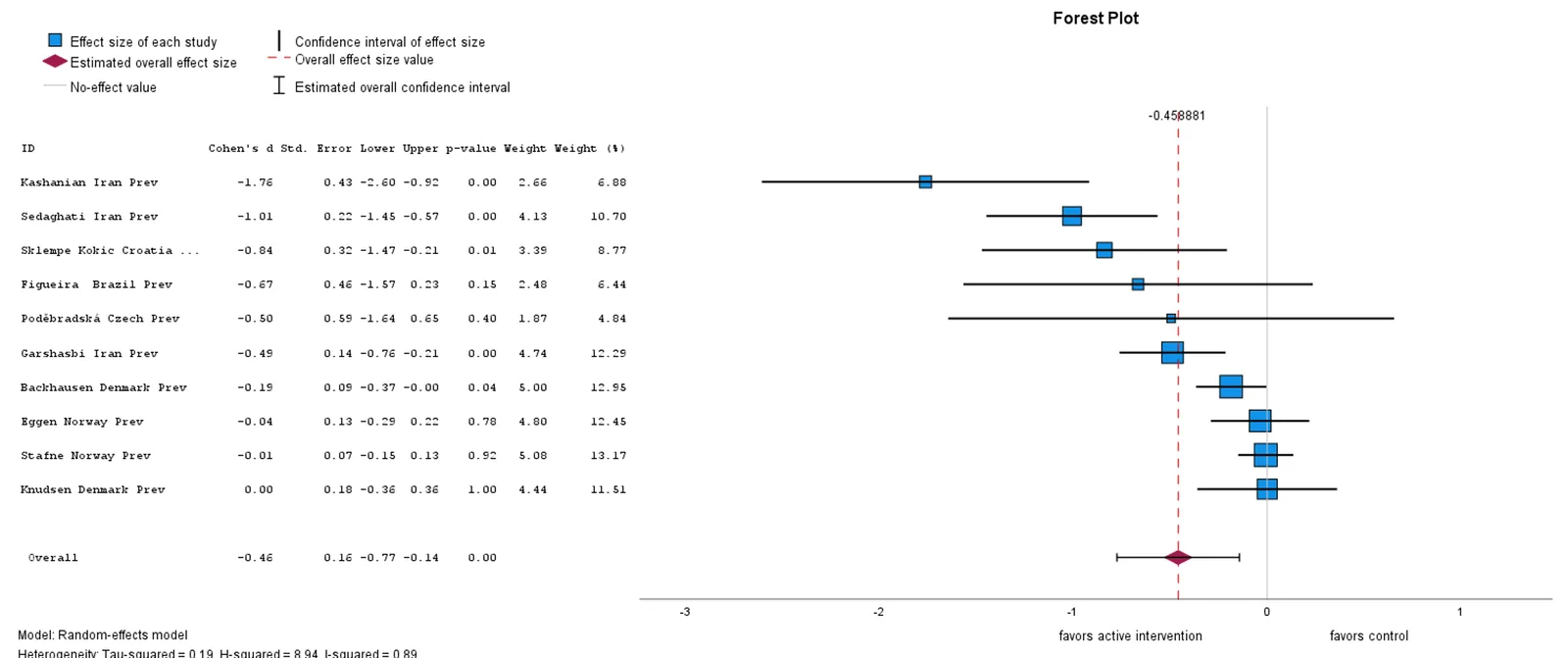
Pain decrease in preventive studies. References: Table 2, line 8.

The effect size for pain in the preventive studies (n = 10) was 0.62 (p = 0.009) (Table 2, line 8; Table 3; Figure 5). In five studies, the effect size did not reach the threshold for statistical significance. Excluding the four Scandinavian studies, the effect size was 0.84 (p < 0.001) (Table 3).

In six preventive studies, numerical data on pain reduction were missing (Table 2, line 9). In five of these studies, the authors indicated that no significant difference in pain reduction was found between the experimental groups and usual care; in the remaining study, no information on the statistical significance of the effect was available. Thus, of the 15 preventive studies, 10 (67%) showed no statistically significant reduction in pain development (Table 2, line 10). In contrast, 20 of the 22 therapeutic studies (91%) showed a significant reduction in pain (Table 2). The difference in the proportion of studies showing a significant effect on pain between therapeutic and preventive studies was statistically significant (p < 0.001).

Of the 10 studies performed in Scandinavian countries, only one (10%) showed a significant effect on pain (Table 2, lines 14 and 15). Of the 27 studies conducted in other countries, all but two [13,30] showed a positive result (93%). The difference in the proportion of studies showing a significant effect on pain between Scandinavian and non-Scandinavian countries was statistically significant (p < 0.001).

**Figure 6:**
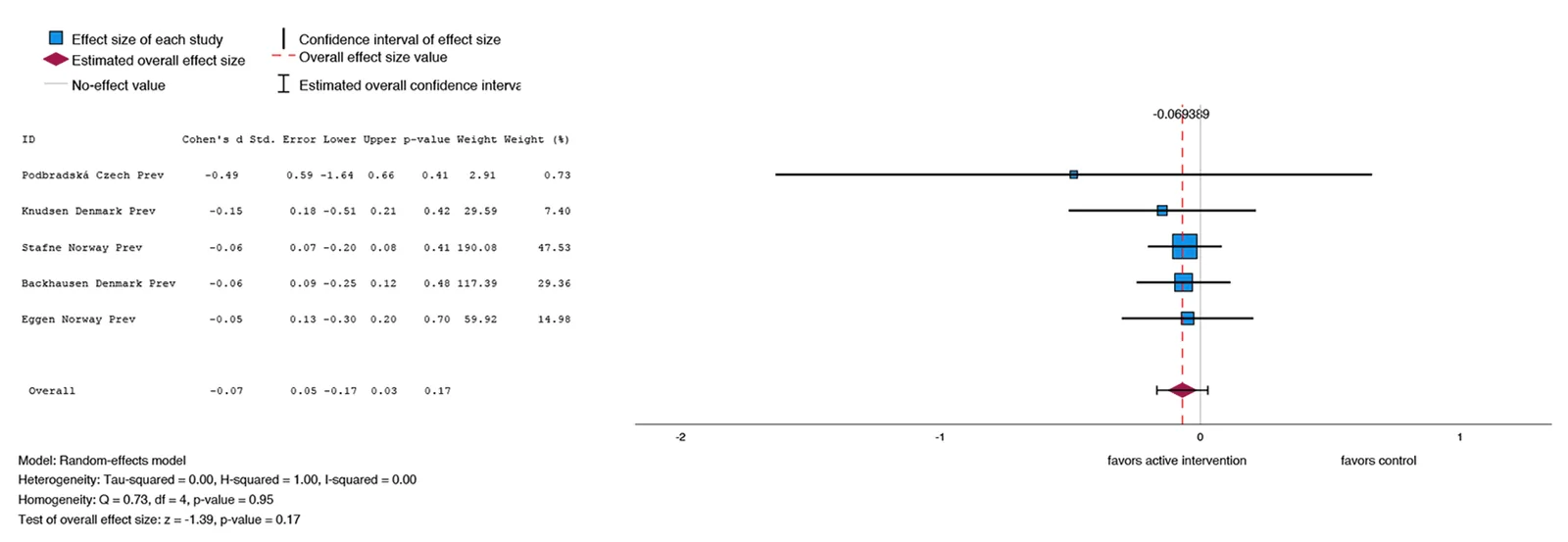
Disability decrease in preventive studies. References: Table 2, line 11.

**Figure 7:**
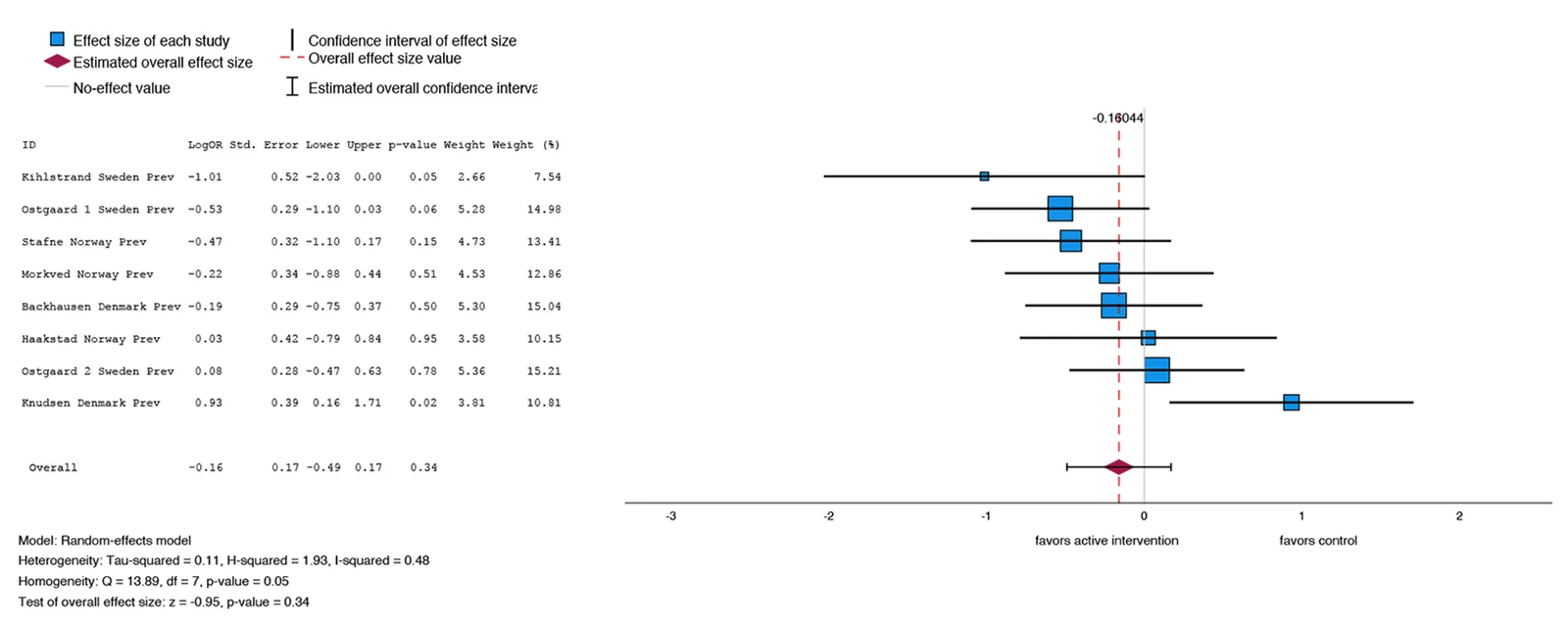
Sick leave percentage in preventive studies. References: Table 2, line 12.

**Figure 8:**
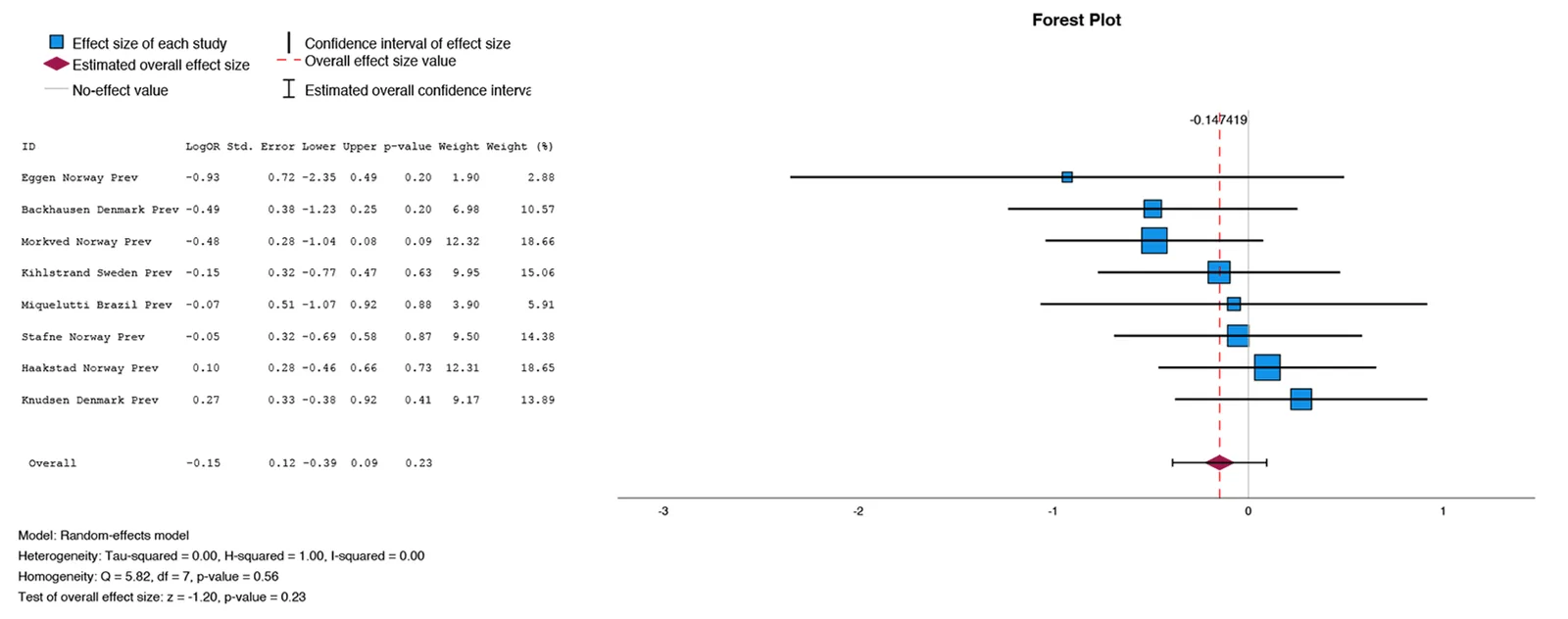
Lumbopelvic pain percentage in preventive studies. References: Table 2, line 13.

With two exceptions, all preventive studies on disability, sick leave, and the proportion of women with LPP were conducted in Scandinavian countries. None of the preventive studies on disability (n = 5), the proportion of women on sick leave (n = 8), or the proportion of women with LPP (n = 8) at the end of pregnancy showed a statistically significant effect (Table 3, lines 11-13; Figures 6-8).

In the experimental groups of the therapeutic studies, mean pain intensity decreased from 5.44 (SD 1.32) at baseline to 2.94 (SD 1.53) at follow-up (p < 0.001). In the preventive studies, pain intensity increased from 1.81 (SD 0.88) to 2.42 (SD 0.70) (p = 0.051). The difference in pain intensity between the preventive and therapeutic experimental groups at baseline was 3.63 (95% CI 2.65 to 4.61; p < 0.001) and at follow-up 0.56 (95% CI −0.23 to 1.36; p = 0.16).

Quality of the Studies

The mean PEDro scores of the Scandinavian and non-Scandinavian therapeutic studies were 6.00 (SD 0.0) and 4.74 (SD 2.10), respectively; the difference between the groups was not statistically significant (p = 0.42). For the preventive studies, the corresponding values were 7.11 (SD 0.93) and 6.00 (SD 1.26), respectively (p = 0.06). No significant association was found between methodological quality and the effect on pain reduction in the 32 studies included in the meta-analysis presented in Figure 2 (β = −0.26, 95% CI −0.82 to 0.27; p = 0.35). The PEDro score explained none of the between-study heterogeneity (R² = 0%).

Heterogeneity and Publication Bias of the Studies

**Figure 9:**
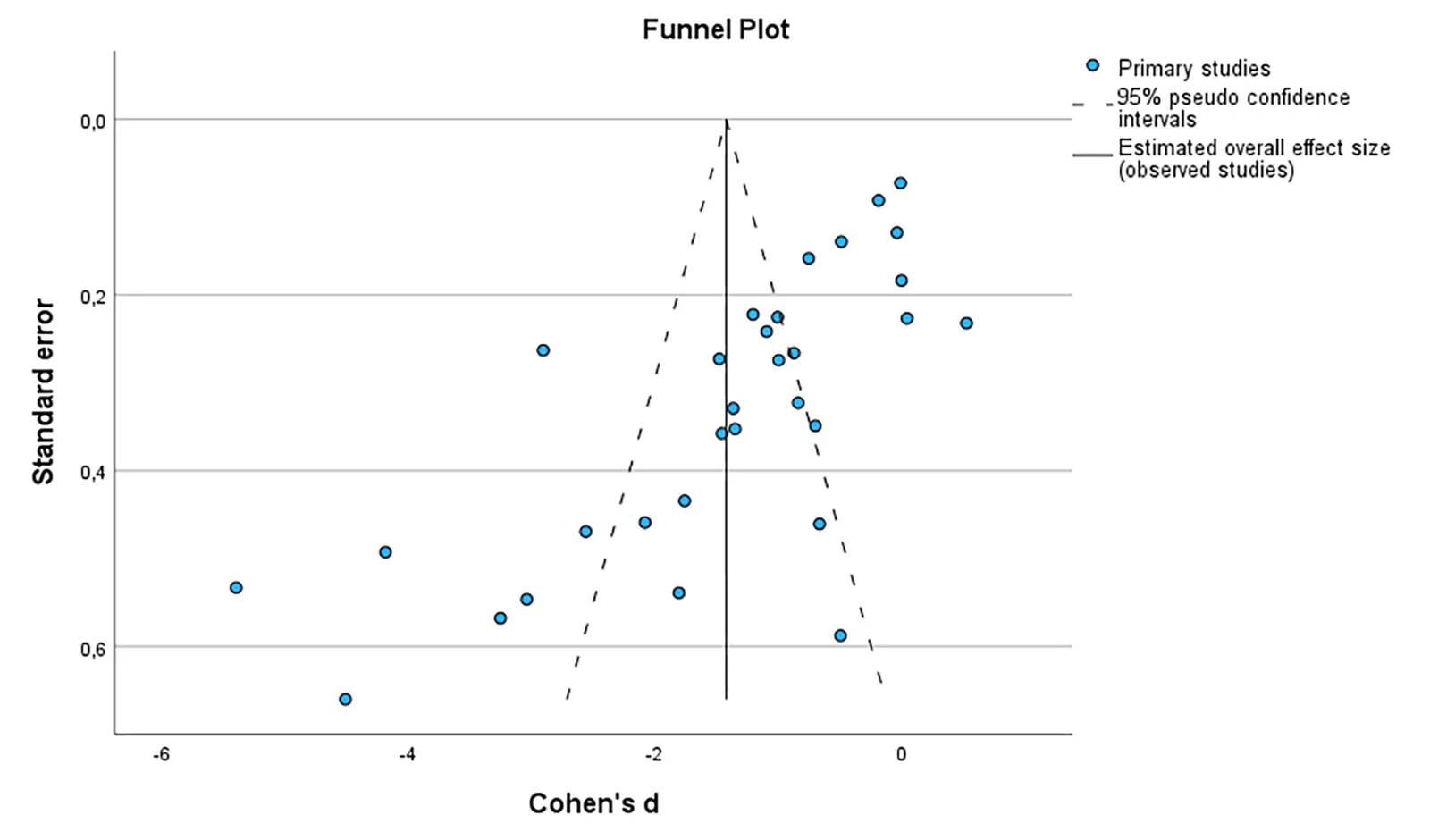
Funnel plot constructed for the meta-analysis of pain outcomes in the therapeutic studies after exclusion of an extreme outlier.

Statistical heterogeneity across the studies, quantified using the I² statistic, ranged from 0 to 0.99 (Table 3). To assess potential publication bias, a funnel plot was constructed for the meta-analysis of pain outcomes in the therapeutic studies after exclusion of the extreme outlier [14] (Figure 9). Egger’s regression test showed no statistically significant evidence of funnel plot asymmetry (intercept = 0.638, p = 0.106).

Nature of Active Interventions

The active interventions varied considerably in content and delivery and included swimming, running, bicycling, aerobics, yoga, Pilates, stretching, and/or strengthening of various muscle groups. In one study, exercises were prescribed to reduce foot pronation (Table 1). The results of the assessment of the components of the active interventions are presented in Tables 4, 5.

**Table 4:** Summary of the various aspects of the interventions in the experimental group of the therapeutic trials. G: Group; H: Home; I: Individually; N: No; N/A: Not Available; Y: Yes.

First author, year of publication	Quality of the trial	Providing Information	Pelvic-stabilizing exercises	Pelvic floor exercises	Cardiovascular training	Number of contacts with health care professional	Individually, Group, or Home	Supervised
Bandpei, 2010 [2]	4	Y	N	N	N	N/A	N/A	Y
Barbier, 2023 [11]	5	Y	N	N	N	1	H	N
Field, 2012 [12]	4	N	N	N	N	24	I	Y
Figueira, 2014 [13]	5	Y	N	N	N	18	N/A	Y
Filipec, 2020 [14]	6	Y	N	N	N	1	H	N
Gil, 2011 [16]	4	N	N	N	N	8	N/A	Y
Keskin, 2012 [18]	4	N	N	N	N	1	H	N
Kluge, 2011 [20]	6	Y	Y	Y	N	6	N/A	Y
Kordi, 2013 [22]	7	Y	N	Y	Y	1	H	N
Mamipour, 2023 [23]	7	N	Y	Y	N	30	G	Y
Martins, 2005 [24]	4	N	N	N	N	10	G	Y
Mirmolaei, 2018 [4]	4	Y	N	Y	N	12	H	N
Niaraki, 2021 [26]	-	N	N	N	Y	24	G	Y
Nilsson-Wikmar, 2005 [27] (Home)	6	Y	N	N	N	2	H	N
Nilsson-Wikmar, 2005 [27] (In Clinic)	6	Y	N	N	Y	32	I	Y
Ozdemir, 2015 [29]	7	Y	N	N	Y	12	I	Y
Ramezanpour, 2018 [31] (Balans)	1	N	N	N	N	36	G	Y
Ramezanpour, 2018 [31] (Kegel)	1	N	N	Y	N	36	G	N
Ramezanpour, 2018 [31] Balans+Kegel)	1	N	N	Y	N	1	H	Y
Sonmezer, 2021 [34]	7	Y	Y	Y	N	16	I	Y
Suputtitada, 2002 [36]	5	N	N	N	N	16	I	Y
Yildirim, 2023 [37]	8	Y	Y	Y	N	16	G	Y

**Table 5:** Summary of the various aspects of the interventions in the experimental groups of the preventive trials. G: Group; H: Home; I: Individually; N: No; N/A: Not Available; Y: Yes.

First author, Year of publication	Quality of the trial	Providing information	Pelvic-stabilizing exercises	Pelvic floor exercises	Cardiovascular training	Number of contacts with health care professional	Individually, Group, or Home	Supervised
Backhausen, 2017 [10]	7	Y	N	N	Y	3	H	N
Eggen, 2012 [6]	6	Y	Y	Y	Y	18	G	Y
Figueira, 2014 [13]	5	Y	N	N	N	18	N/A	Y
Garshasbi, 2005 [15]	8	N	N	N	Y	36	I	Y
Haakstad, 2015 [3]	7	N	Y	Y	Y	30	G	Y
Kashanian, 2009 [17]	6	N	N	N	N	24	H	N
Kihlstrand, 1999 [19]	7	N	N	N	Y	18	G	Y
Knudsen, 2024 [21]	8	N	N	N	Y	45	G	Y
Miquelutti, 2013 [25]	7	Y	Y	Y	Y	11	I	Y
Morkved, 2007 [5]	8	Y	Y	Y	Y	12	G	Y
Ostgaard 1, 1994 [28]	6	Y	N	N	N	2	IH	Y
Ostgaard 2, 1994 [28]	6	Y	N	N	N	5	GH	Y
Poděbradská, 2019 [30]	6	N	N	N	N	1	H	N
Sedaghati, 2007 [32]	4	N	N	N	Y	24	N/A	Y
Sklempe Kokic, 2017 [33]	6	N	N	Y	Y	12	I	Y
Stafne, 2012 [35]	8	Y	Y	Y	Y	12	G	Y

Mode of Usual Care

The included studies were conducted in 12 countries. Information on usual care was available for 10 countries (Table 6). Information about usual care in the Czech Republic and Thailand was unavailable.

**Table 6:** Usual care to treat lumbopelvic pain during pregnancy per country according to literature and personal communication with experts. ^a^ Kurdija K, Gynecologist, University Hospital Centre Zagreb, Croatia (Personal communication, 2024). ^b ^Negahban H, Professor of Physiotherapy, School of Paramedical and Rehabilitation Sciences, Mashhad University of Medical Sciences, Mashhad, Iran (Personal communication, 2023). LBP: low back pain; LPP: Lumbopelvic pain

Country	Authors	Usual care as described by the authors
Scandinavian countries		
Denmark	Backhausen 2017 [10]	Standard prenatal care in Denmark consists of three visits to the general practitioner, five visits to a midwife, and two ultrasound scans. Healthy pregnant women are advised to exercise at least 30 minutes of moderate intensity per day.
Norway	Malmqvist 2012 [38]	19%-52% of patients were treated. 20.6% of all pregnant women exercised >2-3 times a week.
	Gutke 2018 [39]	53% of patients received treatment. 90% of them were satisfied.
Sweden	Gutke 2018 [39]	34% of patients received treatment. 84% of them were satisfied.
	Mogren 2005 [40]	32.7% of patients received some treatment. Women with high pain levels: 51.2%.
Non-Scandinavian Countries		
Brasilia	Martins 2005 [24]	Two of 36 patients were referred to a physiotherapist.
	Figueira 2014 [13]	None of the 20 patients were treated with exercises.
Croatia	Kurdija^a^ 2024	Below 1% of patients get advice to consult a physiotherapist (expert opinion).
France	Ibanez 2017 [41]	3 of 137 women were treated with physiotherapy.
	Barbier 2023 [11]	The usual management focuses on prescribing analgesic medication and sometimes advising physiotherapy, osteopathy, or swimming.
Iran	Ansari 2010 [42]	41% of patients consulted a physician; 8.5% of all patients were treated.
	Negahban^b^ 2023	Therapists in Iran ‘are usually overprotective with these patients and prefer to use more conventional and safe treatment methods in order to prevent unwanted consequences’ (expert opinion).
Republic of South Africa	Hawker 2021 [43]	1 out of 14 women with severe LPP sought help.
Turkey	Sencan 2018 [44]	4.2% of patients received treatment.
	Ozdemir 2015 [29]	None of the 48 patients were referred to a physiotherapist.
	Ozdemir 2015 [29]	Usual care is comprised of movement without straining herself in daily life, forcing herself to rest, taking paracetamol pills, and consulting a physical therapy clinic for LPP if necessary.
	Sonmezer 2021 [34]	Regular prenatal care consisting of routine medical and nursing care, and given education consisting of ergonomic information about activities that exacerbate LBP during daily living and optimal lifting techniques, sitting, standing and sleeping postures.
USA	Gutke 2018 [39]	24% of patients received treatment. 71% of them were satisfied.

Discussion

The findings of this review support our hypothesis that distinguishing between preventive and therapeutic studies and considering differences in usual care largely explains the apparent inconsistencies in the literature.

The results from the Scandinavian countries differed strikingly from those from the other countries. Only 2 of the 27 comparisons between active interventions and usual care in the Scandinavian studies showed a statistically significant effect (Figures 2-8), whereas 38 of the 41 comparisons in the non-Scandinavian studies were statistically significant. This pattern was consistent across pain, disability, and the development of sick leave and/or LPP. The most plausible explanation is that active interventions already form part of usual care in the Scandinavian countries, thereby reducing the contrast between the intervention and control groups.

Although this explanation cannot be confirmed directly from the available data, it is supported by both the descriptions of usual care in the included studies and the additional literature summarized in Table 6. According to these sources, between 19% and 53% of pregnant women with LPP receive treatment in the Scandinavian countries, compared with 0% to 24% in the other countries. This interpretation is further supported by a Danish study in which women in the intervention group exercised 5.5 hours (SD 4.4) per week compared with 5.2 hours (SD 4.0) in the control group (p = 0.57), indicating that women receiving usual care were already highly physically active [10].

The preventive studies also differed markedly from the therapeutic studies. Only 8 of the 31 comparisons in the preventive studies were statistically significant, whereas 32 of the 37 comparisons in the therapeutic studies showed significant effects. Preventive interventions did not significantly reduce disability, sickness absence, or the incidence of LPP. Pain intensity increased in the preventive studies and decreased in the therapeutic studies, resulting in convergence of mean pain intensity between the two groups and no significant difference at follow-up. These findings suggest that active interventions are particularly effective in reducing the severity of established LPP rather than preventing its onset.

A planned analysis to determine which characteristics of active interventions were associated with positive outcomes had to be abandoned because little variation in treatment effect remained after stratifying the studies according to therapeutic versus preventive design and Scandinavian versus non-Scandinavian setting. Consequently, no meaningful comparisons between intervention characteristics could be made.

The remaining question is whether one type of active intervention is more effective than another. RCTs directly comparing active interventions provide no consistent evidence that one approach is superior [27,28,31,45,46]. Although some individual studies reported better outcomes with individually supervised exercise or water-based exercise, these findings were not consistently replicated across studies.

Moreover, many Scandinavian studies effectively compared two active treatment strategies because usual care already included active management. Overall, the available evidence does not support the superiority of any specific active intervention.

The similarity of outcomes across interventions based on very different biomechanical rationales provides little support for the hypothesis that treatment success depends primarily on specific biomechanical mechanisms. Although various biomechanical explanations have been proposed, including improved cardiovascular fitness, muscular stabilization, force closure, exercise performance, and individual supervision, the present review found no consistent evidence that interventions emphasizing these components achieved superior outcomes.

In contrast, several studies suggest that psychosocial mechanisms may contribute importantly to treatment effects [4,20,29,35,47-53]. Active interventions may reassure women that physical activity is safe during pregnancy, reduce fear of movement, improve confidence, and enhance mood and self-efficacy. Educational components, reassurance, relaxation, body awareness, and supervised exercise were repeatedly identified as potentially important therapeutic elements. Taken together, these findings suggest that reassurance and other psychosocial components may represent common therapeutic elements shared by otherwise diverse interventions.

Mechanical changes of the pelvic girdle during pregnancy are well documented. Increased sacroiliac joint mobility and widening of the pubic symphysis are associated with LPP and impaired load transfer between the trunk and legs [54,55]. These changes are therefore likely to contribute to the development of pain and disability. However, the present review suggests that active interventions mainly reduce pain severity rather than prevent the development of LPP. Notably, complete resolution of pain during pregnancy was rare in the included studies, even after active intervention [27,36], suggesting that treatment primarily reduces symptom severity rather than eliminating pregnancy-related LPP. Together, these findings are consistent with the hypothesis that pregnancy-related LPP originates largely from mechanical changes associated with pregnancy, whereas active interventions mainly reduce symptom severity through psychosocial mechanisms rather than reversing the underlying mechanical changes.

If reassurance is indeed an important therapeutic mechanism, this has implications for clinical communication. Explaining symptoms solely in biomechanical terms, particularly using expressions such as “ruptured” or “broken”, may unintentionally reinforce fear and avoidance. Instead, clinicians may benefit from explaining the normal physiological changes that occur during pregnancy in reassuring language, emphasizing that these changes are recognizable, temporary, multifactorial, and generally associated with a favorable prognosis after delivery [56].

Although statistical heterogeneity was very high (I² up to 99%; Table 3), most studies demonstrated treatment effects in the same direction, indicating that the high I² values primarily reflected variation in the magnitude of treatment effects rather than inconsistency in their direction. Differences in usual care, intervention type, study populations, and the wide range of outcome measures used to assess pain and disability are all likely contributors to this heterogeneity. The funnel plot appeared reasonably symmetrical (Figure 9), and Egger’s regression test showed no statistically significant evidence of funnel plot asymmetry. Thus, we found no indication of small-study effects or publication bias.

In summary, distinguishing between preventive and therapeutic studies and accounting for differences in usual care largely resolves the apparent inconsistencies in the literature on active interventions for pregnancy-related LPP. Active interventions appear to be beneficial, particularly for women with established LPP, but no convincing evidence indicates that one active intervention is consistently superior to another. The findings further suggest that the beneficial effects of active interventions may be explained at least as much by reassurance and other psychosocial components as by the specific biomechanical content of the intervention.

Limitations and strengths

The main strength of this review is that it included a relatively large number of studies without restrictions on language, country, or year of publication. The principal limitation was the small number of therapeutic studies conducted in Scandinavian countries, which reduced the statistical power of subgroup analyses. In addition, pain outcomes were assessed using both Numeric Rating Scale (NRS) and Visual Analog Scale (VAS), which were not standardized across studies.

Another limitation concerns the characterization of usual care. Information on usual care was obtained from published literature and personal communications with experts. Consequently, some misclassification of usual care across countries cannot be excluded. However, the high degree of consistency in the findings across studies conducted within the same country suggests that any such misclassification is unlikely to have materially affected the results.

A further limitation is that study selection was initially performed by a single reviewer. Although a second reviewer confirmed the eligibility of the 38 included studies, the complete screening process was not performed independently by two reviewers.

Although the methodological quality of the included studies varied considerably, meta-regression showed no significant association between methodological quality and treatment effect, suggesting that study quality did not materially influence the overall findings.

## Conclusions

We hypothesized that the conflicting findings of studies evaluating active interventions for LPP during pregnancy would be reduced by accounting for variations in usual care and by distinguishing between preventive and therapeutic studies. Our findings support this hypothesis. A marked difference was observed between studies conducted in Scandinavian and non-Scandinavian countries. Although information on usual care was limited, the available evidence suggests that referral to physiotherapy is more common in Scandinavian countries, where usual care generally includes reassurance and exercise therapy, whereas in many other countries it more often consists of advice to reduce activity or passive treatments. These differences may partly explain why randomized controlled trials in Scandinavian countries are less likely to demonstrate additional benefits of active interventions over usual care. The same pattern was observed in both preventive and therapeutic studies, with Scandinavian studies showing smaller effects than non-Scandinavian studies. However, the difference between Scandinavian and non-Scandinavian studies was more pronounced in therapeutic studies than in preventive studies.

No consistent differences in effectiveness were observed between the different types of active interventions. This suggests that intervention-specific biomechanical components may not be the primary determinants of treatment outcome. One possible explanation is that these interventions share common non-specific therapeutic elements, such as reassurance, encouragement to remain active, increased confidence in movement, and other psychosocial factors. However, because no psychosocial outcomes were measured or synthesized in this review, this interpretation should be regarded as a hypothesis rather than a conclusion directly supported by the data. Future studies should investigate the contribution of these potential mechanisms.

## Appendices

Appendix A: Search strategy 

(pregnancy OR gestation OR gravidity OR pregnant OR prepartum) AND (“back pain” OR  “posterior pelvic pain” OR “pregnancy-related pelvic joint pain” OR “pregnancy-related pelvic girdle pain” OR “pelvic girdle pain” OR “peripartum pelvic pain” OR “pelvic girdle relaxation” OR “SI-joint” OR sacroiliac OR “pubic symphysis” OR lumbar OR lumbopelvic OR lumbosacral OR “pelvic ring” OR pelvis OR “pelvic ligaments” OR “lumbar vertebr*”) AND (“randomised controlled trial” OR “randomized controlled trial” OR “randomized control trial” OR RCT OR “clinical trial” OR  “controlled clinical trial” OR “cohort”) AND (prevent* OR  intervention OR treatment OR manipulation OR manipulative OR mobilisation OR mobilization OR “manual therapy” OR “physical therapy” OR “passive move­ment” OR  chiropract* OR osteopath* OR exercise OR training OR relaxation OR stabilization OR stabilisation OR strength OR stabilizing OR stabilising OR stretching OR coordination OR acupuncture OR belt OR garment OR support [ti] OR brace )

(last performed on January 5, 2026)

Appendix B

(pregnancy OR gestation OR gravidity OR pregnant OR prepartum) AND (“back pain” OR  “posterior pelvic pain” OR “pregnancy-related pelvic joint pain” OR “pregnancy-related pelvic girdle pain” OR “pelvic girdle pain” OR “peripartum pelvic pain” OR “pelvic girdle relaxation” OR “SI-joint” OR sacroiliac OR “pubic symphysis” OR lumbar OR lumbopelvic OR lumbosacral OR “pelvic ring” OR pelvis OR “pelvic ligaments” OR “lumbar vertebr*”) AND (name of the country [TI] OR adjective of the name of the country [TI])

(last performed on January 5, 2026)

NB (name of the country [TI] adjective of the country [TI]) means, for example, (Norway [TI] OR Norwegian [TI]).
